# Revisiting small‐molecule fluorescent probes in food freshness detection

**DOI:** 10.1002/smo2.70072

**Published:** 2026-07-15

**Authors:** Xinrui Wang, Zhao Wang, Jingwen Zou, Xuyuan Jiang, Changyu Bian, Zhiqiang Mao

**Affiliations:** ^1^ Ministry of Education Key Laboratory for the Synthesis and Application of Organic Functional Molecules, Hubei Province Key Laboratory of Biotechnology of Chinese Traditional Medicine College of Health Science and Engineering Hubei University Wuhan China; ^2^ Wuhan Business University Hubei Provincial Engineering Research Center of Racing Horse Detection and Application Transformation Equine Science Research and Horse Doping Control Laboratory Wuhan China

**Keywords:** biogenic amines, fluorescent probes, food freshness, smartphone‐based sensing

## Abstract

Food spoilage driven by microbial growth severely endangers public health and global food security. Traditional methods for food freshness evaluation suffer from cumbersome operation, time‐consuming procedures, and poor portability. In contrast, fluorescent probe‐based detection offers distinct advantages including high sensitivity, fast response, and visual detectability. This review summarizes recent advances in organic small‐molecule fluorescent probes for food freshness assessment over the past 3 years. We focus on representative sensing mechanisms of these probes, including nucleophilic substitution, protonation/deprotonation, hydrogen‐bonding interactions, Lewis acid‐base reactions, and Michael addition as well as typical target analytes such as biogenic amines, hydrogen sulfide, sulfur dioxide derivatives, pH, ATP, and hydrazine. The representative applications of fluorescent probes in portable test strips, smartphone‐assisted sensing platforms, and intelligent detection systems are emphatically introduced. Although these probes have achieved remarkable results in sensitivity, response speed and practicality, challenges still exist such as single detection target, insufficient anti‐interference ability, and lack of prospective prediction function. Future research will focus on expanding detection scopes, developing multi‐target recognition probes, combining artificial intelligence to construct spoilage prediction models, and promoting the industrial application of portable and intelligent food freshness detection technology.

## Graphical abstract

Fluorescent probes serve as versatile tools for food freshness monitoring. This review summarizes recent advances in probes targeting biogenic amines, hydrogen sulfide, pH values, and other key spoilage markers, highlighting their design principles, sensing mechanisms, and practical applications in food quality monitoring.

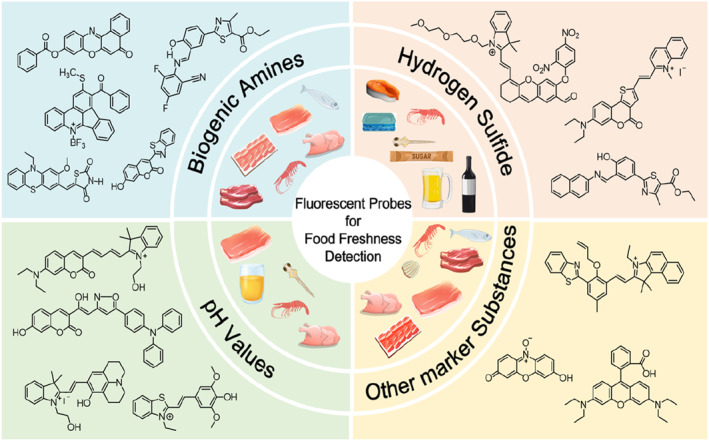

## 1 INTRODUCTION

Currently, global population growth has exacerbated microbial contamination in fresh and processed foods, presenting dual challenges to public health and economic stability via spoilage during processing, storage, and transportation.[^1^, ^2^] Meanwhile, the United Nations Food and Agriculture Organization notes that climate change is modifying the growth patterns of pathogenic microorganisms, thereby amplifying risks to food security.^[3]^ The growth and reproduction of microorganisms, including bacteria and fungi, are the main causes of food spoilage, with their characteristic harmful metabolites widely used as biomarkers to assess food freshness. Traditionally, consumers have evaluated food freshness using sensory indicators such as color, odor, and texture.^[4]^ Nevertheless, these approaches suffer from inherent limitations, including subjectivity and poor quantifiability. To date, researchers have established several effective approaches for detecting food freshness, including high‐performance liquid chromatography,^[5]^ gas chromatography,^[6]^ and spectroscopic analysis.^[7]^ However, these methods are cumbersome, time‐consuming, and highly dependent on experimental equipment and conditions, rendering them unsuitable for routine practical detection.

By contrast, fluorescence probe‐based assay technology‐characterized by high sensitivity, excellent specificity, low toxicity, minimal biological impact, and cost‐effectiveness—has emerged as an innovative approach in food detection.[^8^, ^9^, ^10^, ^11^] Activated fluorescence probes can be rationally designed to respond to various biomarkers of food freshness such as biogenic amines (BAs), hydrogen sulfate, and other related substances. Furthermore, these probes can be integrated into rapid, portable, and miniaturized detection devices (e.g., test strips and smartphone‐based sensors), enabling both destructive and nondestructive food analysis and facilitating the translation of such analytical methods into practical applications.[^12^, ^13^, ^14^, ^15^] Against this background, fluorescence probe‐based assays hold promise in the fast detection of food freshness. So far, there are few reviews that systematically summarize the advancements of fluorescent probes in food freshness detection. Accordingly, this review summarizes the design, applications, and recent advances of organic small‐molecule fluorescent probes in food freshness detection over the past 3 years. We believe this review provides a general framework for the design of fluorescent probes toward food freshness evaluation and will facilitate the development of more probes for practical food detection and food safety assurance.

## 2 BIOMARKER GENERATION MECHANISMS OF FOOD SPOILAGE

**FIGURE 1:**
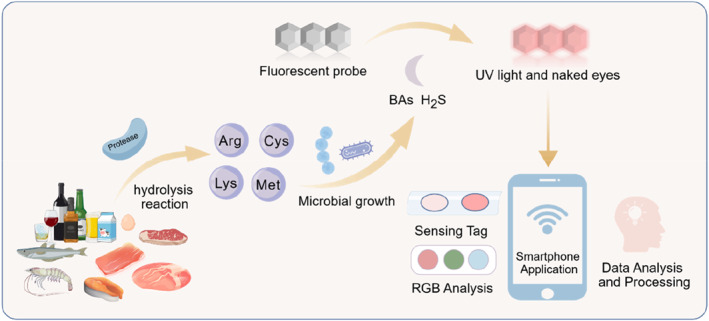
Schematic illustration of fluorescent probes for food freshness detection.

Food spoilage is not caused solely by microbial contamination but rather represents a comprehensive outcome of dynamic interactions and evolution among microorganisms, food matrices, and external environments (e.g., temperature, packaging gas composition, etc.). Biogenic amines, hydrogen sulfide, and other compounds serve as effective biomarkers for evaluating food freshness (Figure 1).[^16^, ^17^]

Biogenic amines are primarily formed via microbial‐mediated amino acid decarboxylation. In animal‐derived food systems such as meat, milk, and dairy products, proteases secreted by microorganisms or endogenous enzymes in food gradually hydrolyze proteins into polypeptides and free amino acids, among which arginine, lysine, ornithine, and others provide key precursor substrates. Under favorable conditions such as anaerobiosis and neutral pH, amine‐producing microorganisms, including lactic acid bacteria and Enterobacteriaceae, activate specific intracellular amino acid decarboxylases (e.g., arginine decarboxylase, lysine decarboxylase, etc.). These decarboxylases catalyze the decarboxylation of the corresponding amino acids to produce carbon dioxide and the respective BAs.[^18^, ^19^, ^20^] The continuous accumulation of BAs in food, once reaching a certain concentration threshold, not only causes off‐odors and quality deterioration but also poses food safety risks and threatens human health.^[21]^

Hydrogen sulfide is mainly generated through microbial‐mediated desulfurization of sulfur‐containing amino acids. Muscle proteins in food matrices are hydrolyzed by proteases secreted by microorganisms such as Shewanella and *Clostridium*, producing sulfur‐containing amino acids including cysteine and methionine as precursors for hydrogen sulfide synthesis. Under suitable environmental conditions including anaerobiosis and neutral pH, hydrogen sulfide‐producing microorganisms activate key intracellular desulfurase systems such as cysteine desulfurase and methionine γ‐lyase, which further catalyze the catabolism of sulfur‐containing amino acids. Specifically, cysteine is converted into pyruvate, ammonia, and hydrogen sulfide via desulfurase catalysis, whereas methionine is mainly degraded by methionine γ‐lyase to produce *α*‐ketobutyrate, methanethiol, ammonia, and a small amount of hydrogen sulfide. Part of the generated hydrogen sulfide is released and accumulated in the food system, exhibiting a characteristic pungent rotten‐egg odor; another part binds to myoglobin in meat to form green sulfmyoglobin, resulting in abnormal green discoloration. These changes collectively serve as important indicators of severe food spoilage.[^21^, ^22^]

Essentially, food spoilage involves microorganisms utilizing nutrients in food for metabolic proliferation and producing characteristic metabolites such as BAs and hydrogen sulfide, ultimately leading to quality deterioration and loss of edibility.[^23^, ^24^] Therefore, the rational design and development of fluorescent probes capable of specifically recognizing biomarkers including BAs and hydrogen sulfide enables real‐time monitoring of the spoilage process and accurate assessment of food freshness.

## 3 TARGET BIOMARKERS

### 3.1 BAs‐based food freshness detection

Biogenic amines are small organic compounds generated primarily via microbial decarboxylation of free amino acids. They are categorized into aliphatic, aromatic, and heterocyclic amines according to their chemical structures. Eight types of BAs are commonly discovered in food including histamine (HIS), putrescine (PUT), tyramine (TYR), cadaverine (CAD), β‐phenylethylamine (PHE), tryptamine (TRY), spermidine (SPM), and spermine.[^25^, ^26^, ^27^] As food spoils, BA concentrations increase markedly, particularly in high‐protein foods such as meat and seafood. These compounds are formed through the decarboxylation of various amino acids by substrate‐specific decarboxylases present in bacteria that thrive in spoiled food. Beyond producing offensive odors, excessive BAs induce adverse physiological effects, including headaches, hypotension/hypertension, nausea, palpitations, and renal toxicity; severe exposure may even cause cerebral hemorrhage or death. According to Chinese national food safety standards, regulatory limits for BAs primarily target histamine—the most toxic BA. Limits vary by food category: Histamine levels were no more than 400 mg/kg for high‐histamine fish, 200 mg/kg for other seafood, and 100 mg/kg for meat and fermented meat products.[^26^, ^28^, ^29^]

**FIGURE 2:**
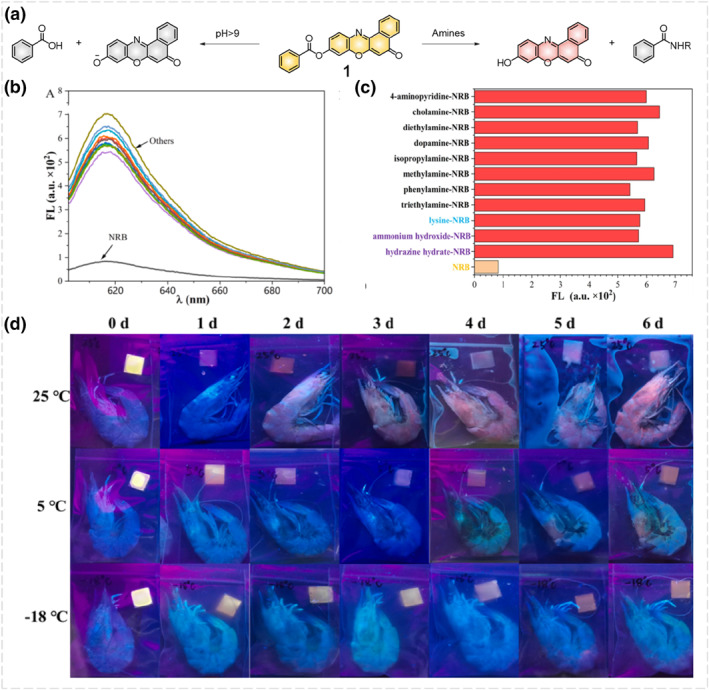
(a) Reaction of probe 1 (NRB) with RNH_2_. (b) Fluorescence responses of probe 1 (2 μM) to various amines in DMSO‐MES. (c) Fluorescence intensities of probe 1 at 615 nm toward different amines. (d) Monitoring shrimp freshness with NRB at different temperatures. Reproduced with permission from ref.^[30]^ Copyright 2023 Elsevier B.V.

Numerous fluorescent probes for food BA detection are designed based on nucleophilic substitution reactions. For instance, in 2023, Wang et al. fabricated a benzoxazine‐based dual‐emission fluorescent probe 1 (NRB) for BA detection to evaluate shrimp freshness (Figure 2a).^[30]^ methylamine (MeNH2), isopropylamine, and other amines act as nucleophiles to attack the benzoate moiety of probe 1 via nucleophilic substitution, cleaving the ester bond and generating corresponding benzamide derivatives alongside de‐esterified fluorescent xanthene fluorophore (NR) with a maximum emission at 615 nm. Experiments also revealed that OH^−^ can induce hydrolysis of the benzoate moiety in probe 1 and enhance its fluorescence under alkaline conditions (pH > 9). Accordingly, amine triggered much stronger fluorescence enhancement of probe 1 at pH 7–9. The probe shows negligible fluorescence responses to other gases (ammonia, hydrogen chloride, hydrogen sulfide) and non‐nucleophilic analytes (Figure 2b,c). Subsequently, probe 1 was further applied for real‐time freshness monitoring of shrimp, exhibiting a distinct yellow‐to‐red color change readily identifiable by the naked eye (Figure 2d). Additionally, the probe enables ultra‐sensitive colorimetric (50 ppt, naked‐eye visible) and NIR fluorescent detection of BAs in both aqueous solution and gas phases rapidly (<30 s), featuring dual gas–liquid applicability and an ultralow detection limit (50 ppt for MeNH2 by naked eye and 17 ppt by fluorescence spectrometer, respectively).

**FIGURE 3:**
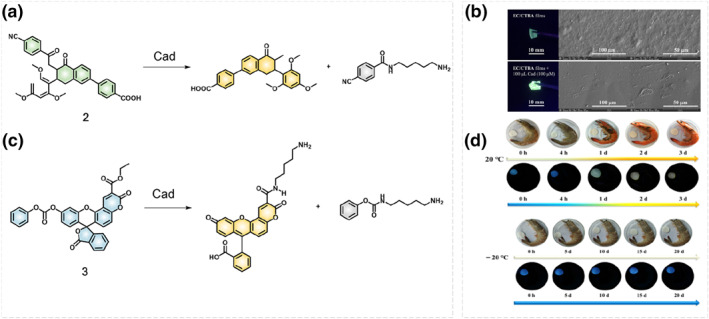
(a) Reaction of probe 2 (CTBA) with cadaverine. (b) Fluorescence and SEM images of ethyl cellulose (EC)/CTBA films before and after treatment with Cad. (c) Sensing mechanism of probe 3 (SWJT‐35) toward biogenic amines (BAs). (d) Schematic of noncontact, nondestructive shrimp freshness detection. Reproduced with permission from ref.^[31]^ Copyright 2025 Elsevier B.V.; ref.^[32]^ Copyright 2025 Elsevier B.V.

Although probe 1 (NRB) has enabled real‐time fluorescence visualization of shrimp spoilage, recent efforts have focused on developing new skeleton‐based probes with improved portability and quantification ability.[^31^, ^32^] In 2025, Huang et al. constructed a flavonol cyanobenzyl ester‐based “turn‐on” fluorescent probe 2 (CTBA) for BAs in fish and prawn via nucleophilic substitution.^[31]^ CTBA exhibited outstanding analytical performance, with a detection limit (limit of detection (LOD)) of 3.60 × 10^−7^ M toward Cad, a rapid response within 20 min at 25°C, and excellent operational stability over a wide pH range of 4–10.^[30]^ The response mechanism of probe 2 toward BAs could be described as follows. Biogenic amines (e.g., cadaverine, putrescine, spermine) attack and cleave the ester bond of probe 2 (CTBA), releasing the fluorophore and enhancing the intramolecular charge transfer (ICT) effect to produce strong yellow‐green fluorescence (Figure 3a). Furthermore, probe 2 was self‐assembled with ethyl cellulose (EC) via hydrogen bonding to prepare composite fluorescent films. Upon exposure to 100 μL of 100 μM cadaverine (Cad), the EC/CTBA film exhibited a color transition from pale cyan to bright yellow under 365 nm UV irradiation. SEM images revealed local aggregation and structural shrinkage of the EC/CTBA film, enabling efficient capture and detection of the cadaverine (Figure 3b). This strategy expands its practical applications in seafood freshness evaluation.

Very recently, Du and co‐workers synthesized a ratiometric fluorescent probes 3 (SWJT‐35) bearing coumarin‐fluorescein hybrid skeletons for food‐related Cad detection, with low detection limits of 0.17 μM (visible light) and 0.23 μM (fluorescence) for cadaverine (Cad).^[32]^ Probe 3 reacts with Cad through nucleophilic substitution at the carboxylic ester and carbonate groups, producing dual‐color fluorescent and colorimetric signals (F_549_/F_439_) (Figure 3c). Moreover, probe 3 was fabricated into portable test‐strip labels. Combined with smartphone‐based RGB analysis, these labels realized non‐contact, non‐destructive, and real‐time monitoring of shrimp freshness (Figure 3d). The smartphone‐based RGB analysis method provides a rapid, facile, and accessible approach for daily food freshness detection, holding great practical promise for market applications.

**FIGURE 4:**
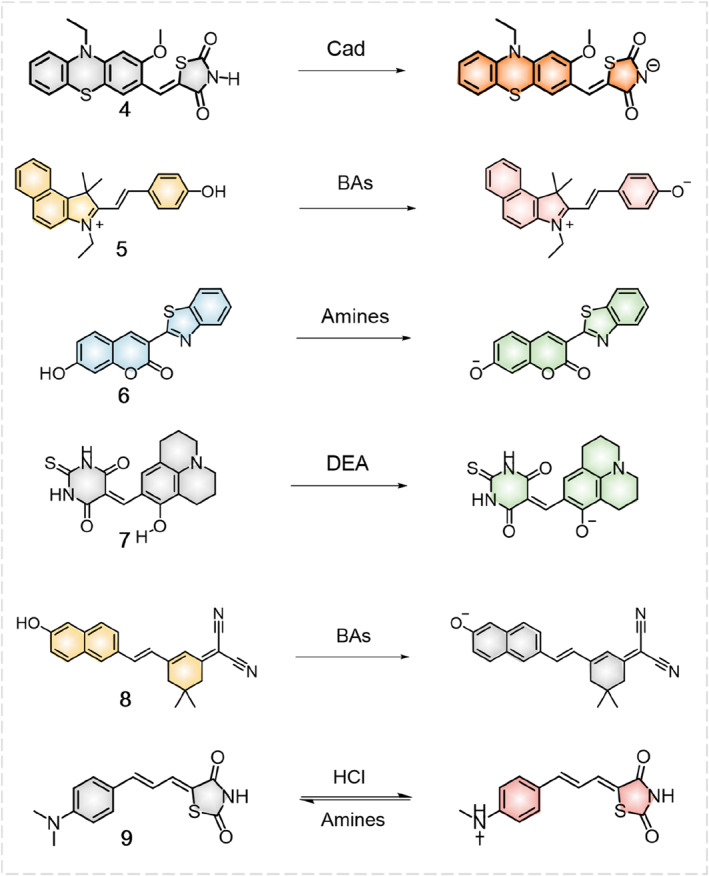
Reaction of probe 4 (MPZ) with cadaverine. Reaction of probe 5(BCY‐OH) with biogenic amines (BAs). Reaction of probe 6 (MUS) with amines. Reaction of probe 7 (HTBR) with DEA. Reaction of probe 8 (HYM) with BAs. Reaction of probe 9 (CDT) with amines.

**FIGURE 5:**
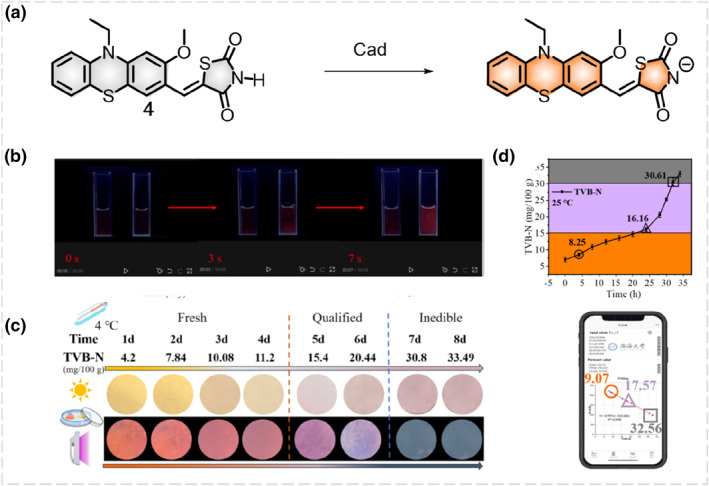
(a) Reaction of probe 4 (MPZ) with cadaverine. (b) Fluorescence color evolution of MPZ with Cad over time. (c) Color changes of MPZ/FPS under daylight and UV light for 8 d. (d) TVB‐N values from standard method and intelligent platform. Reproduced with permission from ref.^[33]^ Copyright 2025 Elsevier B.V.

Aside from nucleophilic substitution reactions, fluorescent probes based on protonation/deprotonation mechanisms have also been extensively developed for food freshness detection (Figure 4). In 2025, Sun's lab reported a phenothiazine‐based “OFF–ON” fluorescent probe 4 (MPZ) for fish freshness evaluation via a deprotonation mechanism, which exhibits remarkable advantages in the detection of BAs in EtOH/H_2_O (4/6, *v/v*) solution, including rapid response speed (within 7 s), low detection limit (0.72 μM), and a distinct red emission signal with a maximum emission wavelength of 620 nm.^[33]^ BAs activates the probe via deprotonation, suppresses the photoinduced electron transfer (PET) effect, and triggers an OFF‐to‐ON fluorescence turn‐on response (Figure 5a). Notably, the probe achieves rapid dual colorimetric/fluorescent signal responses within 7 s (Figure 5b). Furthermore, probe possesses excellent reversible sensing performance; upon introducing HCl to trigger reprotonation, the system can undergo repeated OFF–ON–OFF switching cycles. Probe 4 was loaded onto filter paper to construct MPZ/FPS sensing tags, which display distinct fluorescence color changes under daylight and 365 nm UV irradiation (Figure 5c). A supporting intelligent application named “Visual Evaluation” was further developed. The quantitative values calculated by the APP are highly consistent with those measured using national standard methods, enabling non‐destructive, rapid, and instrument‐independent intelligent assessment of fish freshness (Figure 5d).

Apart from acidic N–H bonds, other alkali‐sensitive groups in fluorophores, such as phenolic hydroxyl (Ar–OH) moieties, can be engineered to detect food‐derived amines. In 2025, Miao et al. designed a donor–π–acceptor colorimetric and fluorescent probe 5 (BCY‐OH) for BAs in food via the deprotonation of the phenol unit.^[34]^ In the presence of cadaverine, the phenolic hydroxyl group of BCY‐OH undergoes deprotonation to form a phenoxide anion, enhancing the ICT and fluorescence emission, while producing a distinct color change (from green to purple) within 16 s (0–54.4 ppm, LOD = 0.18 μM).

**FIGURE 6:**
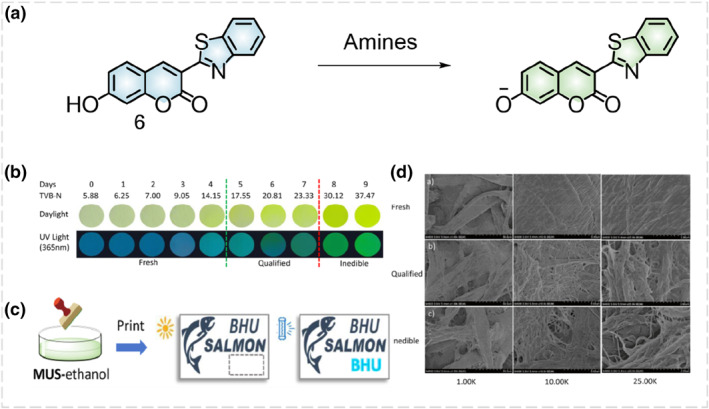
(a) Reaction of probe 6 (MUS) with amines. (b) Color changes of labels and corresponding TVB‐N values. (c) Schematic of anti‐counterfeiting label using probe 6 (MUS) ink. (d) SEM images for fish freshness grading with varied magnifications. Reproduced with permission from ref.^[35]^ Copyright 2025 Elsevier B.V.

Similarly, in 2025, Liu et al. developed a benzothiazole‐coumarin‐derived fluorescent probe 6 (MUS) for BAs to evaluate salmon freshness.^[35]^ BAs deprotonate the phenolic hydroxyl group of probe 6 to generate phenoxide anions, resulting in the enhancement of ICT. Correspondingly, the fluorescence shifts from blue to green, enabling selective responses to various BAs (Figure 6a). A smartphone‐assisted dual‐channel (colorimetric/fluorescent) sensing platform was further constructed for accurate freshness evaluation of salmon. In addition, the probe was extended as a fluorescent ink for anti‐counterfeiting printing applications (Figure 6b,d). Meanwhile, in 2025, Zhao's group synthesized a series of fluorescent probes, among which probe 7 (HTBR) with a julolidine skeleton for diethylamine (DEA) (LOD = 2.66 μM).^[36]^ Owing to their alkalinity, engineered BAs (e.g., DEA) can deprotonate the phenolic hydroxyl group of HTBR to form phenoxide anions, completing the response within 2 s. The fluorescence quantum yield increased from 16.93% to 40.77%. In 2025, Yang et al. reported a naphthol–malononitrile‐based fluorescent probe 8 (HYM) for detecting BAs, which showed a detection limit of 4.23 μM for putrescine. The sensing mechanism relies on deprotonation of the phenolic hydroxyl group in HYM, which induces distinct absorption and fluorescence spectral changes.^[37]^ The probe exhibits dual colorimetric and fluorescent responses toward BAs, achieving simultaneous color and fluorescence signal changes within 3 s. Further, the author also prepared a probe‐based non‐contact sensing membrane (HYMPF) for BAs. In 2025, Sun et al. constructed a deprotonation‐driven D‐π‐A fluorescent probe 9 (CDT). Relying on an “OFF–ON” ICT mechanism, the probe responds to 15 kinds of BAs within 5 s. After being immobilized on filter paper, it forms a dual‐channel indicator label for amines. With the assistance of RGB analysis, the label enables real‐time quantitative detection of TVB‐N values in salmon, thereby evaluating fish freshness. Unlike conventional food freshness sensors based on chemical reactions, sensors relying on protonation‐deprotonation reactions feature rapid response and recyclability owing to the fast and reversible nature of these reactions. This type of probe holds great promise for developing rapid detection mini‐devices and enables fast screening of real food samples.

**FIGURE 7:**
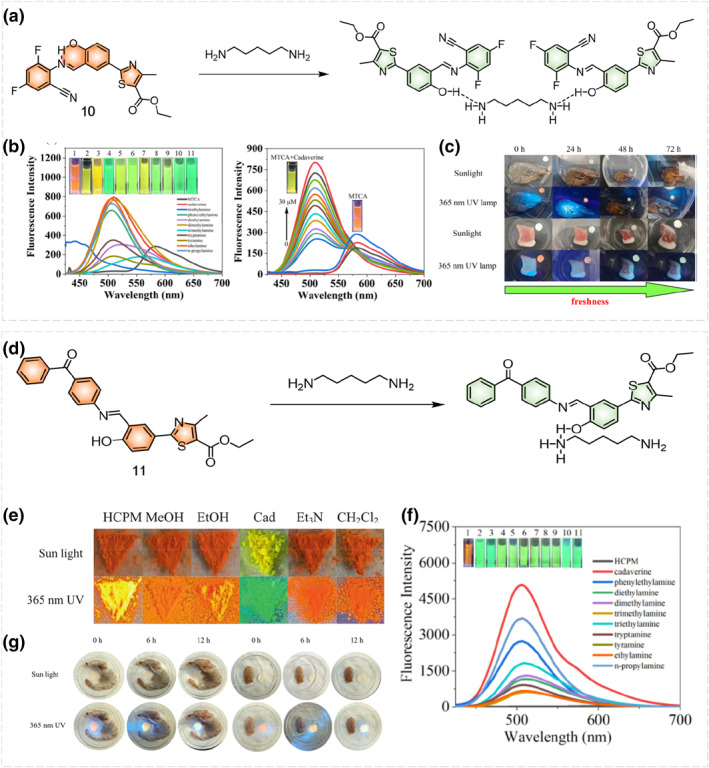
(a) Reaction of probe 10 (MTCA) with cadaverine. (b) Emission spectral changes of probe 10 (MTCA) (10 μM) with cadaverine in EtOH/H_2_O (1/9, *v/v*, pH 7.4). Inset: UV (365 nm) color changes before/after adding cadaverine. (c) Shrimp/pork freshness monitoring with test strips (0–72 h, 365 nm, RT). (d) Reaction of probe 11 (HCPM) with cadaverine. (e) Probe 11 (HCPM) color changes under 365 nm UV (λ_ex_ = 400 nm, slits = 5 nm/5 nm). (f) HCPM (10 μM) fluorescence spectra toward various biogenic amines (BAs) (EtOH/H_2_O 9/1, *v/v*). Inset: UV (365 nm) color changes (1–11 as listed). (g) Pork/shrimp freshness monitoring with test strips (0–12 h, 365 nm, RT); orange‐yellow: fresh, green: slight spoilage. Reproduced with permission from ref.^[38]^ (Copyright 2025 Elsevier B.V.) and ref.^[39]^ (Copyright 2025 Elsevier B.V.).

Beyond the two classic response mechanisms outlined above, intra‐ and intermolecular hydrogen‐bonding competition and conformational regulation have emerged as novel design strategies for fluorescent probes targeting BAs. Since 2025, Li's group has successively developed two aggregation‐induced emission (AIE)‐active fluorescent probes, namely probe 10 (MTCA) and probe 11 (HCPM), for cadaverine detection via hydrogen‐bonding interactions.[^38^, ^39^] The amino groups of BAs (e.g., cadaverine) form new hydrogen bonds with the hydroxyl moieties of the probes, disrupting the original intramolecular hydrogen‐bond network and triggering intramolecular C=N isomerization. This process induces a blue shift in fluorescence emission accompanied by a color change from orange‐red to yellow‐green, thereby achieving synchronous colorimetric and fluorescent dual‐channel responses. Both probes recognize BAs by disrupting intramolecular hydrogen bonding and have been assembled into smartphone‐assisted solid‐state sensing platforms. Benefiting from structural optimization through the introduction of a triphenylmethane amine moiety, probe 11 (HCPM) exhibits an improved LOD as low as 0.45 nM in solution, substantially enhancing its sensing sensitivity. Compared with probe 10, this sensor offers superior practical applicability and on‐site adaptability, facilitating rapid quantitative detection of cadaverine vapor in the solid state within a defined response time. These results demonstrate the evolving potential of AIE probes in intelligent food freshness monitoring. By combining reversible hydrogen‐bond recognition with smartphone RGB analysis, they have established a portable, real‐time, and non‐destructive platform for food freshness evaluation (Figure 7).

**FIGURE 8:**
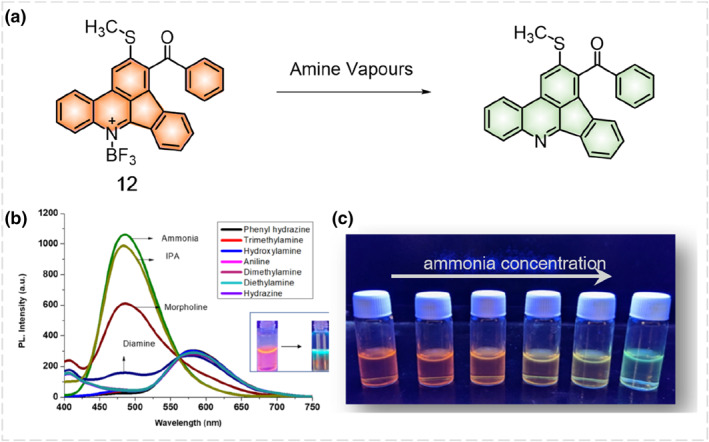
(a) Sensing mechanism of probe 12 (2a) toward amines. (b) Emission spectra of 2a (5 × 10^−5^ M) with various amines (30 μL) in ACN (RH = 65%). (c) Red‐to‐green color transition with increasing ammonia concentration. Reproduced with permission from ref.^[40]^ Royal Society of Chemistry 2026.

Some studies utilize Lewis acid–base interactions and introduce Lewis adduct dissociation mechanisms for amine detection. Unlike conventional sensing pathways such as ester aminolysis, deprotonation, and hydrogen‐bond disruption, this strategy opens a new avenue for amine sensing. For example, Jamuna et al. designed the indophenanthridine derivatives 2a‐c based on nitrogen‐boron Lewis adduct interactions for BA detection.^[40]^ Among them, probe 12 (2a) leverages the photophysical properties of Lewis acid‐base adducts. Specifically, amines remove the nitrogen‐boron moiety of the red‐emissive probe 12 to generate a green‐emissive product (Figure 8a). The probe exhibits highly selective ratiometric fluorescence responses (I_580_/I_480_) toward aliphatic amines. Acting as Lewis bases, aliphatic amines undergo strong ligand‐exchange interactions with the BF_3_ moiety, breaking the N‐B bond and triggering adduct dissociation. Consequently, the fluorescence emission undergoes a blueshift accompanied by a gradual color transition from red to green (Figure 8b,c). In addition, this system permits detection of volatile aliphatic amines down to 22 nM. The authors further constructed a portable test‐strip sensing platform and applied it to the freshness monitoring of fish, chicken, and mutton. This work opens a new direction for the application of Lewis‐adduct‐based fluorescent probes in intelligent food freshness detection. Featuring ultrafast response(<1s), an ultra‐low LOD, and excellent humidity resistance, such probes are particularly suitable for on‐site rapid screening and portable detection scenarios.

Of particular note is that most of the aforementioned fluorescent probes feature a dual‐channel response mode integrating both colorimetric and fluorescent signals. This dual functionality allows visual screening and accurate instrument‐based quantification within a single molecular system, affording self‐calibration, self‐validation, and strong anti‐interference performance in a single molecule entity.

Specifically, several representative probes have been developed to realize such dual‐channel responses with distinct characteristics. For example, Wang et al. reported probe 1 (NRB) for the ultra‐sensitive colorimetric and near‐infrared fluorescence detection of BAs (e.g., 50 and 17 ppt for MeNH2 by naked eyes and fluorescence spectrometer, respectively).^[30]^ Sun et al. developed an “OFF‐ON” probe 4 (MPZ) with a dual‐channel response (colorimetric + fluorescence) within 7 s^[33]^ Du's group designed a coumarin‐based fluorescence ratio‐type probe 3 (SWJT‐35) with dual‐channel fluorescent and colorimetric response.^[32]^ Similarly, Liu et al. developed probe 6 (MUS), achieving “daylight + fluorescence” dual‐channel smartphone‐assisted detection within 11 s^[35]^ Zhao et al. designed probe 7 (HTBR), which provides a dual‐channel response (colorimetric + fluorescence) to BAs within 2 s^[36]^ In addition, Yang et al. developed BA probe 8 (HYM), which exhibits both color and fluorescent changes within 3 s.^[37]^

Moreover, several fluorescent probes exhibit reversible regeneration capabilities, significantly enhancing their reusability and practical application potential. Specific research examples are as follows: Madhuparna et al. designed TCNA, which forms reversible ammonium salts with putrescine vapor and carboxyl groups.^[41]^ Acid vapor treatment can reverse the salt formation, thereby realizing probe regeneration. Sun et al. developed MPZ, which undergoes reversible deprotonation and reprotonation upon interaction with amines and HCl, respectively, enabling reversible OFF–ON–OFF fluorescence switching.^[33]^ Wang et al. synthesized BPS‐A, which relies on amine‐induced reversible “aggregation‐de‐aggregation,” rendering the disruption and reconstruction of π‐π stacking. Its fluorescence color spontaneously recovers upon amine volatilization.^[42]^ Chen et al. designed QF‐G, which relies on reversible Michael addition between amines and its quinolinium salts moiety.^[43]^ After amine volatilization, the conjugated skeleton and dual‐channel signals are fully restored, allowing multiple cycles of use without the need for re‐synthesis. These cyclic regeneration properties not only enhance the longevity of the probes but also increase their efficiency and cost‐effectiveness in practical applications, making them highly suitable for real‐time and repeated monitoring in various fields, such as food quality control and safety detection.

Additionally, several reported fluorescent probes have already realized intelligent and integrated functionality via smartphone applications or other compact electronic devices, markedly enhancing the portability, real‐time capability, and field applicability of food analysis. For instance, Sun et al. developed the “Visual Evaluation” Android/iOS APP, which utilizes the intrinsic fluorescence system of the probe.^[33]^ This APP supports a one‐click workflow, including image capture, RGB value extraction, and freshness level output within 5 s. It supports the automatic output of a three‐level spoilage index with an error of less than 5%. Furthermore, Du et al. integrated the ratio‐type probe SWJT‐35 onto flexible PET microporous membranes, creating a disposable portable test strip label.^[32]^ Coupled with smartphone‐enabled RGB analysis, this system enables real‐time, non‐invasive monitoring of amine vapors inside packaged goods under cold‐chain conditions (0–4°C). The detection limit reaches as low as 0.3 ppm, with a relative standard deviation below 3%. We believe that AI‐powered models can further accelerate the efficiency of smartphone‐based analysis, rendering this approach highly promising for the on‐site, rapid, and reliable monitoring application of food freshness under real‐world conditions.

### 3.2 Biogenic sulfur compounds‐based food freshness detection

**H**_**2**_ **S‐based food freshness detection**: Hydrogen sulfide (H_2_S), as one of the primary volatile spoilage markers of spoiled food, has a significant correlation with food freshness. As anaerobic spoilage bacteria (e.g., sulfate‐reducing bacteria) metabolize sulfur‐containing amino acids such as cysteine and methionine, the released H_2_S can exceed the sensory threshold, thus generating a pungent “rotten egg” odor.[^8^, ^44^, ^45^] Additionally, exposure to H_2_S, whether at low or high levels, poses a hazard to human health. Overall, excessive H_2_S in food not only impairs the taste and texture of food but also presents a potential food safety risk. Therefore, the rapid and on‐site detection of H_2_S in food samples is of particular importance.

**FIGURE 9:**
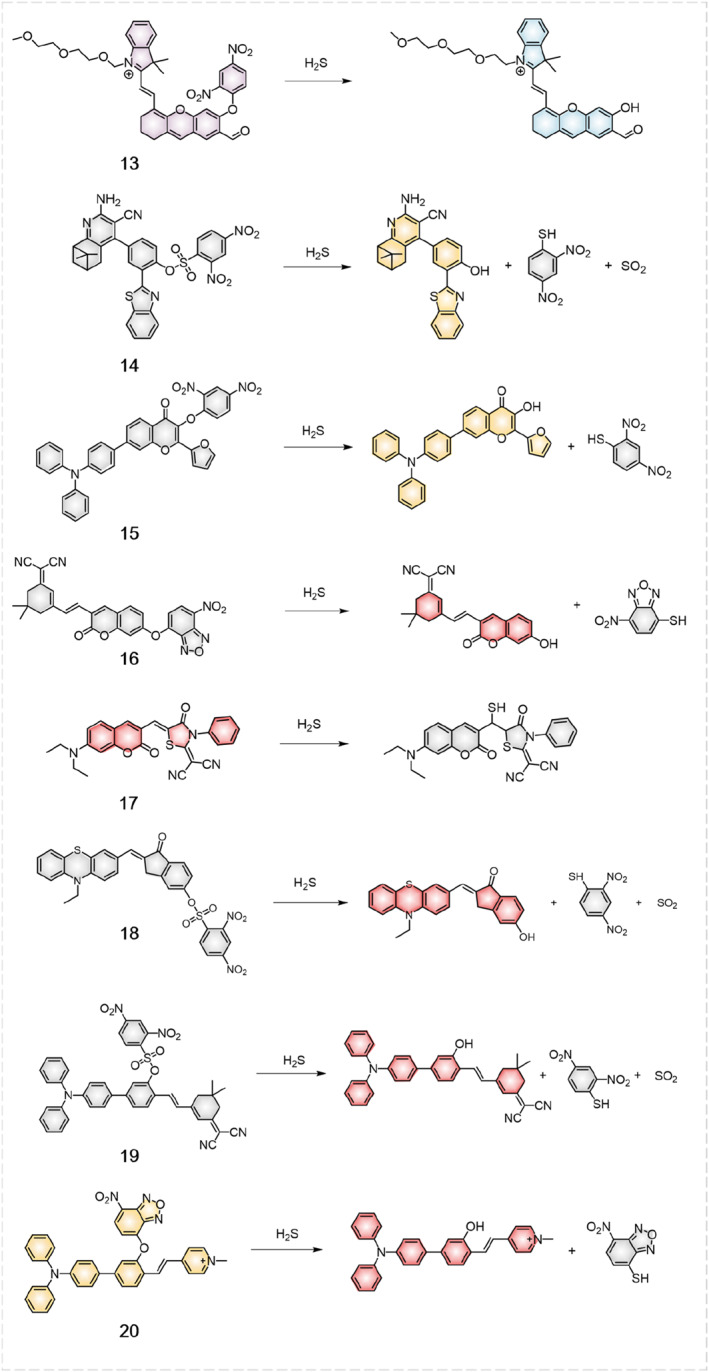
Reaction of probe 13(CHO‐OH‐NO_2_) with H_2_S. Reaction of probe 14 (NPS) with H_2_S. Reaction of probe 15 (DPF‐NP) with H_2_S. Reaction of probe 16 (DEM‐H_2_S) with H_2_S. Reaction of probe 17 (XDS) with H_2_S. Reaction of probe 18 (WX‐HS) with H_2_S. Reaction of probe 19 (TPAT) with H_2_S. Reaction of probe 20 (TPAW) with H_2_S.

**FIGURE 10:**
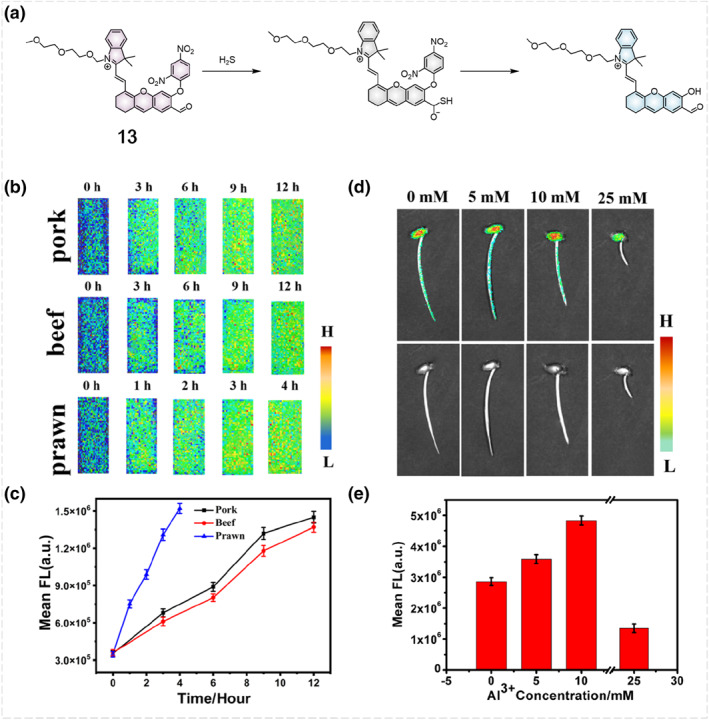
(a) Reaction of probe 13 (CHO‐OH‐NO_2_) with H_2_S. (b) Fluorescence images of test strips for three raw meats at 30°C over time. (c)Average fluorescence intensities corresponding to (b). (d) Fluorescence and brightfield images of wheat seedlings under different Al^3+^ conditions (30°C, 48 h). (e) Mean fluorescence intensities in wheat seedlings (*n* = 5, mean ± SD). Reproduced with permission from ref.^[46]^. Copyright 2023 Elsevier B.V.

In nucleophilic aromatic substitution, HS^−^ (hydrosulfide ion) acts as a potent nucleophile that attacks electron‐deficient aromatic rings or reactive functional groups (e.g., nitro, sulfonyl) in probe molecules. This nucleophilic reaction cleaves ether, sulfonate ester, or sulfonamide bonds, thereby releasing the fluorophore and producing an “off‐on” fluorescence response (Figure 9). For example, in 2023, Liang and coworkers created a NIR fluorescent probe 13 (CHO‐OH‐NO_2_), which achieved the “turn‐on” detection of H_2_S through a tandem nucleophilic addition–intramolecular thiolysis reaction (Figure 10a).^[46]^ The recognition motif takes advantage of the dual nucleophilic properties of H_2_S. HS^−^ first acts as a nucleophile to undergo nucleophilic addition with the aldehyde group to form thiol adduct intermediate. Then, the resulting thiol intermediate then triggers specific cleavage of the dinitrophenyl (DNP) ether bond via intramolecular nucleophilic aromatic substitution. This reaction enhances the ICT effect and leads to a remarkable fluorescence enhancement. Furthermore, the probe 13 was modified with hydrophilic triethylene glycol groups, ensuring stable performance in aqueous‐rich media and enhancing its environmental applicability. In practical applications, the average fluorescence intensity of test strips loaded with probe 13 increases gradually with prolonged storage time, and a faster intensity increase was observed for shrimp samples, confirming that H_2_S can serve as an effective biomarker for evaluating the freshness of high‐protein foods such as seafood and meat (Figure 10b,c). Additionally, the fluorescence intensity of probe 13 initially increases and then decreases with increasing Al^3+^ concentration, reaching a maximum at 10 mM (Figure 10d,e). Further investigations reveal a nonlinear relationship in biological systems: endogenous H_2_S levels in wheat seedlings initially increase and subsequently decrease with increasing plant stress severity, allowing H_2_S to serve as an indicator for evaluating plant stress tolerance. Overall, probe 13 expands its application from food freshness monitoring to plant physiological stress studies, overcoming the single‐scenario limitation of conventional probes and highlighting the versatility and interdisciplinary potential of fluorescent sensors.

**FIGURE 11:**
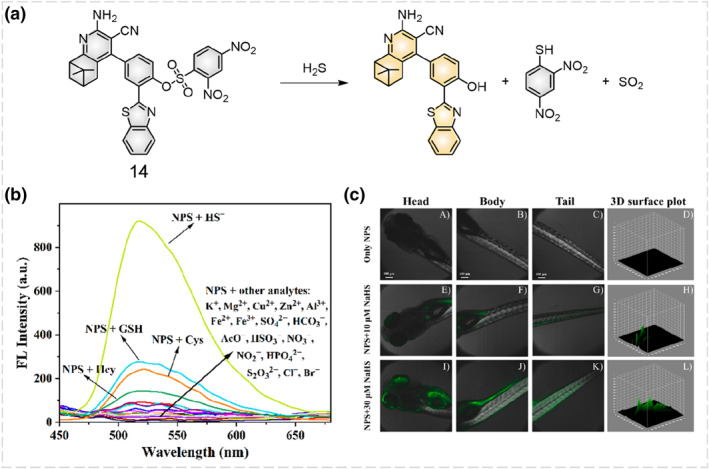
(a) Probe 14 (NPS) reacted with H_2_S. (b) Fluorescent spectra of 10 μM probe 14 (10 mM, pH = 7.4) with various analytes. (c) Confocal fluorescence images and 3D surface plots of HeLa cells: (A−D) 10 μM probe 14 (30 min); (E−H) +10 μM NaHS (30 min); (I−L) +30 μM NaHS (30 min). λ_ex_ = 405 nm, λ_em_ = 500−550 nm, Scale bar = 20 μm. Reproduced with permission from ref.^[47]^ American Chemical Society 2023.

In 2022, Li et al. developed a novel turn‐on fluorescent probe 14 (NPS) for H_2_S detection based on its reaction with sulfonate esters (Figure 11a).^[47]^ Acting as a nucleophile, HS^−^ specifically attacks the carbon atom attached to the sulfonate group of the 2,4‐dinitrobenzenesulfonate moiety, triggering cleavage of the sulfonate bond. As a result, H_2_S removes the PET quencher, *viz*., 2,4‐dinitrobenzenesulfonate moiety, and activates the excited state intramolecular proton transfer (ESIPT) pathway, producing strong yellow fluorescence. Selectivity experiments confirmed that probe 14 displays excellent specificity toward H_2_S, effectively discriminating it from biological thiols (GSH, Cys) and other common interferents, thus enabling accurate detection in complex sample matrices (Figure 11b). Three‐dimensional imaging clearly visualizes the spatial distribution of probe 14 fluorescence intensity in the head, trunk, and tail regions of zebrafish. With favorable biocompatibility and superior in vivo imaging performance, probe 14 facilitates concentration‐dependent monitoring of exogenous H_2_S in zebrafish (Figure 11c). Practical application evaluations demonstrate that probe 14 not only enables quantitative determination of H_2_S in real water, alcoholic beverages, and egg samples, but also permits fluorescence imaging of exogenous H_2_S in living cells and zebrafish, with applications spanning environmental, food, and biomedical fields. From molecular design and in vitro characterization to in vivo imaging and real‐sample analysis, probe 14 establishes a complete technical platform for high‐performance H_2_S fluorescent probe development, providing a solid experimental basis for its broad application in environmental monitoring, food safety, and biomedical imaging.

Probes 14–19 further exemplify H_2_S‐responsive fluorescent probes based on nucleophilic reactions, applicable to food freshness evaluation. Briefly, in 2023, Wang's group constructed an ESIPT‐based “off–on” fluorescent probe 15 (DPF‐NP) with a flavonol scaffold and an ultra‐large Stokes shift of 210 nm for H_2_S detection.^[48]^ HS^−^ induces nucleophilic thiolysis of the DNP ether linkage and cleaves the bond, resulting in approximately a 130‐fold fluorescence enhancement. The probe enables rapid (3 min), highly sensitive (LOD = 96 nM), and highly selective detection of H_2_S with resistance to thiol interference. In 2024, Chen et al. proposed a coumarin‐derivative NIR probe 16 (DEM‐H_2_S).^[49]^ Similar to probe 14, HS^−^ cleaves the diphenyl ether moiety in probe 15, activating ESIPT, ICT, and concomitant fluorescence. In 2024, Shang et al. developed a D‐π‐A type coumarin‐based red fluorescence probe 17 (XDS), which uses the C=C double bond as a reaction site to interrupt the ICT effect, achieving “ON‐OFF” fluorescence quenching.^[50]^ In 2024, Xu et al. reported a near‐infrared fluorescent probe 18 (WX‐HS) for H_2_S detection based on a phenothiazine scaffold.^[51]^ HS^−^ triggers nucleophilic hydrolysis at the DNBS (2,4‐dinitrobenzenesulfonate) moiety, releasing SO_2_ and turning on the ICT process, thereby achieving near‐infrared emission at 660 nm with an extremely large Stokes shift of 220 nm. The probe exhibited a response time of only 90 s and a detection limit as low as 0.14 μM. In 2025, Tan's group reported a triphenylamine‐based NIR fluorescent probe 19 (TPAT) in which HS^−^ can attack a sulfonate ester, leading to the release of a phenolic hydroxyl group, accompanied by the restoration of both ICT and AIE.^[52]^ Its strong anti‐interference capacity in plant tissues provides new perspectives for agricultural product analysis. In 2025, Huang et al. developed a near‐infrared fluorescent probe 20 (TPAW), which underwent HS^−^‐induced thiolysis of an NBD ester to release the fluorophore TPAW‐OH, generating an “off‐on” fluorescence enhancement within 10 s^[53]^ This probe combines three key advantages including mitochondrial targeting, NIR emission, and ultrafast response.

**FIGURE 12:**
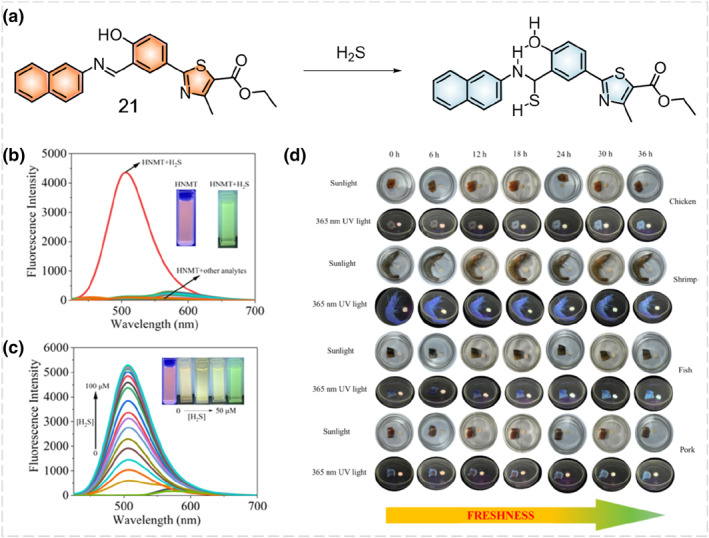
(a) Reaction of probe 21 (HNMT) with H_2_S. (b) Fluorescence responses of probe 21 (HNMT) toward various analytes. Inset: Visual color change under 365 nm UV light before and after H_2_S addition. (c) Emission spectral changes of probe 21 (HNMT) with titration of H_2_S. (d) Photographs of chicken, shrimp, fish and pork under daylight and 365 nm UV light. Reproduced with permission from ref.^[54]^. Royal Society of Chemistry 2025.

Recently, Li et al. reported AIE fluorescent probe 21 (HNMT) for H_2_S sensing via triple synergistic interactions.^[54]^ In contrast to conventional nucleophilic aromatic substitution or Michael addition pathways, its H_2_S sensing proceeds via three synergistic processes: nucleophilic addition at the C=N double bond, rearrangement of the intramolecular hydrogen‐bond network, and modulation of the AIE aggregation state. Specifically, H_2_S (HS^−^) attacks the C=N carbon of probe 21 disrupting its intramolecular hydrogen‐bond network. This induces an aggregation‐state conformational transition of the AIE‐active probe (HNMT), leading to a distinct fluorescence color switch from orange‐red to green with a low detection limit (0.35 μM) (Figure 12a). Furthermore, the key sensing parameters of probe 21 (HNMT) for H_2_S detection, including selectivity and sensitivity, and a linear detection range of 0–50 μM, were systematically characterized, establishing a solid experimental basis for its practical applications in food freshness monitoring and bioimaging (Figure 12b,c). In practical applications, portable fluorescent test strips based on HNMT have been successfully used for real‐time freshness evaluation of various meats, including chicken, shrimp, fish, and pork (Figure 12d). Meanwhile, the probe enables precise fluorescence imaging of both exogenous and endogenous H_2_S in living cells. These results fully illustrate the practical utility and cross‐scenario adaptability of such unconventional AIE probes, providing a novel reference for mechanistic innovation in gas‐sensing fluorescent probes.

**FIGURE 13:**
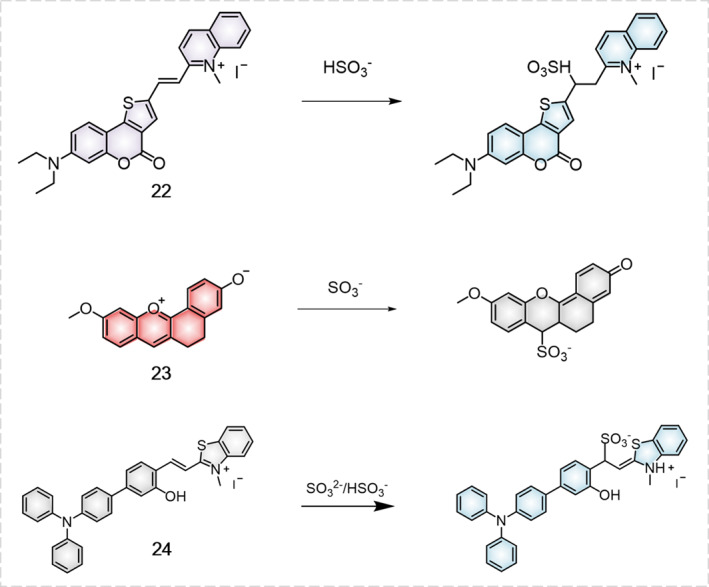
Reaction of probe 22 (SL) with HSO_3_^−^. Reaction of probe 23 (Hy‐S) with SO_3_^−^. Reaction of probe 24 (ZR‐I) with SO_3_^2−^/HSO_3_^−^.

**SO**_**2**_ **‐based food freshness detection**: The sensing mechanism of SO_2_‐activated probes typically involves Michael addition between the C=C double bond and SO_2_ derivatives (SO_3_^2−^, HSO_3_^−^), disrupting the probe's π‐conjugated system and altering its fluorescence (Figure 13).[^55^, ^56^] HSO_3_^−^ is predominantly detected under acidic conditions, while SO_3_^2−^ mainly exists in alkaline environments. They are two existing forms of sulfur dioxide in aqueous solution, and their relative proportions depend on the pH value of the system.

In 2023, Shang et al. developed a coumarin‐based fluorescent probe 22 (SL). The electron‐deficient C=C double bond in the probe molecule undergoes a Michael addition reaction with SO_2_ derivatives (HSO_3_^−^) to form the adduct SL‐SO_3_H, enabling ultrafast (40 s), ultrasensitive (detection limit as low as 38 nM) and highly selective detection of SO_2_ derivatives.^[57]^ As the reaction proceeds, the fluorescence color of the probe solution changes from blue‐violet to light blue, facilitating visual identification of SO_2_ derivatives. Furthermore, this probe has been successfully applied to the detection of residual SO_2_ derivatives in real food samples such as canned goods, sugar, and wine, demonstrating excellent practical application value. In 2025, Huang's group designed a cyaninium‐based probe 23 (Hy‐S) for the detection of free sulfur dioxide and total sulfur dioxide in beer, realizing dual‐functional detection using a single probe for the first time.^[58]^ Under both acidic (pH = 4.0) and alkaline (pH = 9.0) conditions, probe 21 undergoes a Michael addition reaction with HSO_3_^−^/SO_3_^2−^, which disrupts its conjugated skeleton, blocks the ICT process, and consequently induces fluorescence quenching with the disappearance of green emission. This probe exhibits selective responses to SO_2_ under both pH conditions, with detection limits of 0.076 mg·L^−1^ (acidic) and 0.041 mg·L^−1^ (alkaline), and response times within 1 min (acidic) and 30 s (alkaline), respectively. This probe not only broadens the pH range applicable for sulfur dioxide detection but also provides a new tool for the discrimination and rapid screening of sulfur dioxide species in foods, especially beer. Different from probe 21 which exhibits a turn‐off fluorescence response toward SO_2_, probe 24 (ZR‐I) shows an opposite turn‐on fluorescence behavior upon interaction with SO_2_ derivatives. In 2025, Xu et al. constructed a D‐π‐A conjugated structure probe 24 (ZR‐I) based on triphenylamine derivatives, which is used for the rapid detection of sulfur dioxide derivatives (SO_3_^2−^/HSO_3_^−^) in food, environmental samples, and biological systems.^[59]^ In the presence of SO_3_^2−^/HSO_3_^−^, their strong nucleophilicity drives a Michael addition reaction with C=C bonds, disrupting the π‐conjugated system, inhibiting the free rotation of single bonds, and leading to a significant enhancement of fluorescence (the fluorescence of probe 24 (ZR‐I) is enhanced by about 1500 folds) with a detection limit of 0.10 μM. This probe can realize multi‐scenario imaging in water, food, living cells and zebrafish, and can be integrated with a smartphone platform for convenient on‐site detection.

**FIGURE 14:**
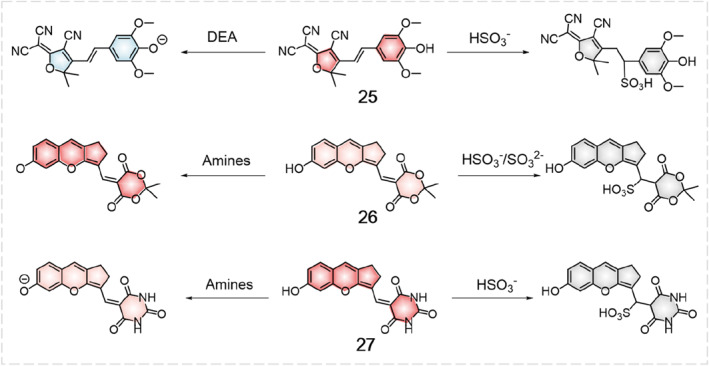
Dual‐functional small‐molecule fluorescent probes for the recognition of SO_2_ derivatives and BAs. Reaction of probe 25 (Sycy) with HSO_3_^−^ and DEA. Reaction of probe 26 (Dpyt) with HSO_3_^−^/SO_3_^2−^ and amines. Reaction of probe 27 (HDXM) with HSO_3_^−^ and amines.

**SO**_**2**_ **‐mediated multi‐target food freshness detection**: Probes enabling multi‐target detection provide extensive information on food quality and represent an attractive developmental trend in food freshness assessment. For instance, dual‐function probes for SO_2_ derivatives and BAs have been designed, offering versatile detection capabilities. In 2023 and 2024, Zhong's lab sequentially designed NIR fluorescence probe 25 (Sycy) and D–π–A‐type probe 26 (Dpyt) for the dual detection of BAs and HSO_3_^−^ (Figure 14).[^60^, ^61^] Probe 25 exhibits fluorescence quenching at 650 nm toward HSO_3_^−^ within 150 s and responds within 5 s to ester bond alkaline hydrolysis induced by DEA, presenting colorimetric‐fluorescent dual‐channel detection of 13 volatile amines in salmon. Analogously, probe 26 (Dpyt) furnishes orthogonal detection of HSO_3_^−^ and organic amines. Upon reaction with HSO_3_^−^, it exhibits fluorescence quenching at 658 nm within 5 s, whereas deprotonation of its phenolic hydroxyl group allows specific recognition of organic amines accompanied by fluorescence enhancement at 660 nm. Probe 26 (Dpyt) has also been further deployed to quantitative detection of HSO_3_^−^ in red wine and white sugar as well as intracellular imaging studies.

In 2024, Yang et al. developed a benzopyran derivative probe 27 (HDXM), for the quick detects SO_2_ derivatives and BAs.^[62]^ HSO_3_^−^ induces fluorescence quenching of probe 27 at 630 nm within 3 s. Meanwhile, it recognizes BAs via phenolic hydroxyl deprotonation accompanied by fluorescence enhancement at 635 nm. This probe affords a “turn‐off + turn‐on” dual‐channel signal output, rendering it promising for multifunctional applications including sensor tags, hydrogels, and fluorescent inks. It has been employed for real‐time assessment of salmon freshness using a dual‐channel visual and smartphone‐based analytical system. These dual‐functional probes markedly improve the speed, specificity, and versatility of food freshness detection, offering new possibilities for real‐time, on‐site applications.

### 3.3 pH value‐based food freshness detection

**FIGURE 15:**
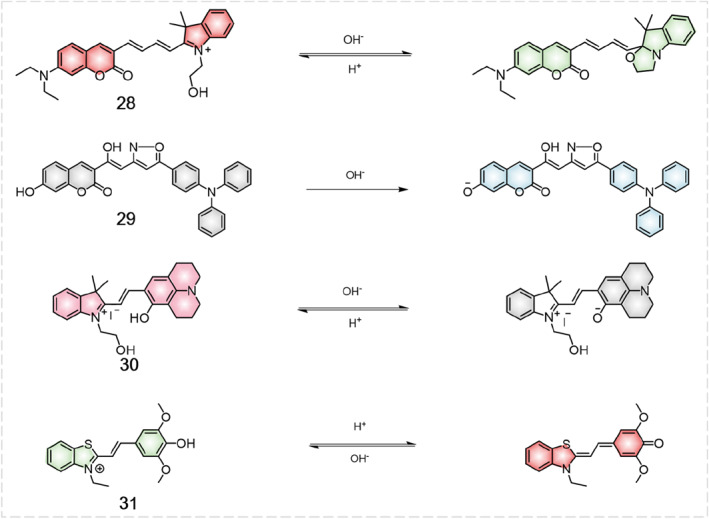
Sensing Mechanism of Probe 28 (Hx) to pH. Sensing Mechanism of Probe 29 (DIC) to pH. Sensing Mechanism of Probe 30 to pH. Sensing Mechanism of Probe 31 (Mi‐Ly‐1) to pH.

In addition to typical food spoilage markers such as BAs, H_2_S, and sulfur dioxide derivatives, pH value fluctuations in food matrices also act as a critical indicator for evaluating food freshness.[^63^, ^64^, ^65^] During microbial proliferation and metabolism, the accumulation of acidic or alkaline metabolites can significantly alter the pH of food systems. Therefore, real‐time and sensitive monitoring of pH changes provides a simple and effective strategy for judging food spoilage and quality. In recent years, pH‐responsive fluorescent probes have been widely explored for food freshness detection due to their fast response, high sensitivity, and straightforward visualization. Representative advances in this field are summarized below (Figure 15).

In 2023, He et al. designed and synthesized a novel colorimetric–near‐infrared fluorescent probe 28 (Hx), which enabled highly sensitive detection of subtle pH fluctuations (0.1–0.2 pH units) in the near‐neutral range in food matrices such as cantaloupe, watermelon, and peach juice, with a response time of less than 10 s^[66]^ Under weakly alkaline conditions (pH > 7.17), intramolecular nucleophilic addition of the phenolic hydroxyl group to the indolium C=N^+^ occurs and forms a five‐membered ring, resulting in the disruption of the molecular conjugated system and inhibition of the ICT effect, and the probe exhibits green fluorescence (λ_em_ = 521 nm). In contrast, under weakly acidic conditions (pH < 7.17), the nucleophilicity of the hydroxyl group is weakened, the cyclic structure undergoes reversible ring‐opening, the conjugated system and ICT effect are restored, and the probe emits near‐infrared fluorescence (λ_em_ = 727 nm). In 2024, Liu et al. constructed a reversible turn‐on fluorescent probe 29 (DIC) with a pH response range of 6.50–9.98, and its response mechanism was deprotonation‐induced fluorescence enhancement.^[67]^ Under weakly acidic to neutral conditions (pH < 8.12), the hydroxyl group exists in a protonated form, resulting in weak fluorescence of the probe. Under alkaline conditions (pH > 8.12), the hydroxyl group undergoes deprotonation to generate an oxygen anion (–O^−^), which significantly enhances the ICT effect, leading to a remarkable increase in the probe fluorescence, and the solution changes from colorless to cyan.

Similarly, in 2024, Zhang et al. synthesized a hemicyanine dye probe 30, which enabled food freshness monitoring based on protonation/deprotonation.^[68]^ The probe exhibited a pH response range of 3.0–9.0 with a linear response interval of 5.0–7.0. It showed fluorescence turn‐on under acidic conditions and fluorescence turn‐off under alkaline conditions, accompanied by a visual color change of the solution from purplish red to pink. In 2026, Huang's group designed and synthesized a novel D–π–A structured fluorescent probe 31 (Mi‐Ly‐1), which integrated ultrahigh pH sensitivity and dual‐targeting imaging capabilities.^[69]^ Upon deprotonation under alkaline conditions, its electron‐donating ability was significantly enhanced, accompanied by an obvious spectral red shift. In the pH range of 4.0–8.5, the absorption ratio (A_550_/A_420_) increased by 80‐fold with increasing pH, demonstrating excellent pH responsiveness. Meanwhile, the probe achieved dual‐color and dual‐targeting imaging of mitochondria and lysosomes at the single‐molecule level, effectively avoiding spectral crosstalk and tedious operations associated with traditional multi‐dye co‐staining. Furthermore, this probe could accurately determine the pH values of foods such as watermelon and cantaloupe, and could also be fabricated into portable test strips for on‐site monitoring of seafood freshness, greatly expanding the application scope of pH fluorescent probes in the field evaluation of food quality.

In summary, pH‐responsive fluorescent probes enable convenient, real‐time food freshness evaluation by sensitively detecting pH changes from microbial metabolism. These probes feature fast response, good reversibility and high sensitivity, and show great potential in portable and multi‐scenario applications.

### 3.4 Other marker substances for freshness detection

**FIGURE 16:**
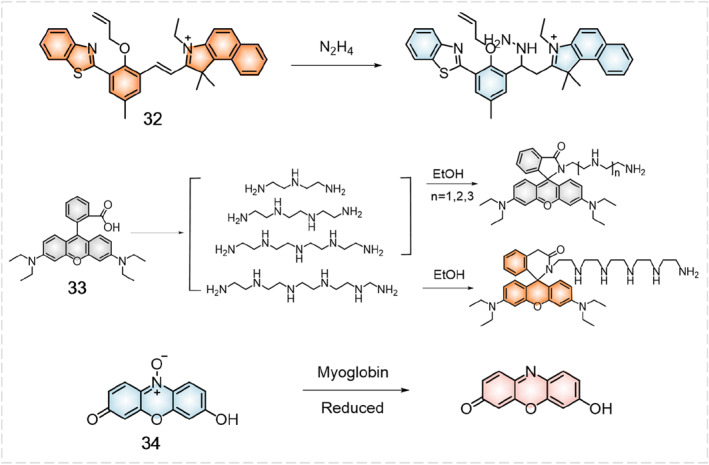
Reaction of probe 32 with N_2_H_4_. Reaction of probe 33 (RP) with ATP. Reaction of probe 34 (Resazurin) with myoglobin (Fe^2+^).

Recently, in addition to well‐known hydrogen sulfide, SO_2_ derivates, and BAs, other biogenic species—including hydrazine, ATP, malachite green, and iron valence changes in myoglobin—can also be detected and employed as food freshness markers.^[70]^ For instance, in 2025, Yang et al. developed a water‐soluble benzothiazole‐benzindole conjugated probe 32 that recognizes hydrazine via 1,4‐addition to the C=C bond driven by its high nucleophilicity (Figure 16).^[71]^ Within 30 min, hydrazine covalently binds to the probe scaffold, yielding a 7.16‐fold fluorescence enhancement at 486 nm. This probe enables the sunlight‐fluorescence dual‐channel visualization detection of hydrazine in water, soil, plants, and river shrimp spoilage. In 2024, Su et al. synthesized a water‐soluble cationic probe 33 (RP) via amide coupling of Rhodamine B with hexaethylene heptamine (PEHA) for ATP detection in various meat.^[72]^ Negatively charged ATP binds to the multiple positively charged amino groups of probe *33* (RP) via electrostatic and π‐π stacking interactions, triggering spirolactam ring‐opening and fluorescence activation at 580 nm. ATP can be detected with the probe in 10 min. This probe has been used for on‐site ATP detection on pork, beef, and other meat surfaces, showing high correlation (*R* = 0.95) with commercial BacTiter‐Glo™ kits and good synchronization with TVC/TVB‐N freshness indicators. Of note, this work provides a rapid, cost‐effective, and visual approach for evaluating meat microbial contamination, expanding the toolkit for food freshness detection. In 2025, Zhu et al. developed a myoglobin–sword blue redox colorimetric system in which Fe^2+^‐myoglobin reduces the biocompatible blue redox indicator sword blue to pink trialling, enabling 30‐min visualization of meat (pork, beef, chicken) freshness via smartphone RGB thermal imaging.^[73]^ The results agreed well with TVB‐N and UV–Vis measurements and were unaffected by common amino acid interferences. This method enables rapid, low‐cost, instrument‐free monitoring of meat freshness for household applications. These studies illustrate the growing versatility of detection probes, employing new freshness markers for real‐time, cost‐effective, and non‐invasive food quality and safety evaluation.

## 4 SUMMARY AND OUTLOOK

This review provides a detailed summary of the research progress and applications of small molecular fluorescent probes for food freshness detection in the past 3 years. These probes have shown significant advantages in detecting spoilage markers such as BAs and hydrogen sulfide (Table 1). Firstly, these probes feature fast response times and ultra‐sensitive detection capabilities, with limits of detection reaching down to the ppt, nM, or μM ranges. Secondly, they have multi‐phase detection abilities, making them adaptable to various environments such as gas phase and liquid phase. A few probes can achieve reversible cycle regeneration through acid treatment, protonation, or deprotonation, thus broadening the scope of detection. Moreover, the applications of these probes are diverse. For instance, they have been extended from meat products, seafood, and wine freshness detection to environmental monitoring (e.g., water samples, soil pollution assessment) and biological imaging (e.g., cell imaging), providing visual tools for a variety of testing scenarios. Additionally, these probes demonstrate strong practical utility, as they can be readily fabricated into sensor films, hydrogels, and fluorescent tags for on‐site and portable detection. Some probes can even be processed into fluorescent inks, offering additional functions such as anti‐counterfeiting and information storage.

**TABLE 1:** Analytical performance of fluorescent probes for food freshness detection.

Probe	Analyte	Sensing mode	Fluorescent color change	Linear range	λ_ex_/λ_em_ (nm)	Response time	LOD
1 (NRB)	Amines (methylamine)	Colorimetric and turn‐on fluorescence	Yellow → pink	0–15 ppm	580/615	≤30 s	17 ppt (fluorescence); 50 ppt (naked eye)
2 (CTBA)	Biogenic amines (cadaverine, putrescine)	Turn‐on fluorescence	Weak cyan → bright yellow green	0–45 μM (Cad)	365/510	20 min	0.36 μM (cadaverine)
3 (SWJT‐35)	Cadaverine	Colorimetric and ratiometric fluorescence	Blue → bright yellow	N.A.	330/439	≤17 min	0.17 μM (fluorescence); 0.23 μM (UV–Vis)
4 (MPZ)	Biogenic amines	Colorimetric and fluorescent (OFF‐ON)	Weak fluorescence → orange‐red	0–50 μM (Cad)	410/620	7 s	0.72 μM
5 (BCY‐OH)	Biogenic amine	Colorimetric	Yellow → purple	0–36 μM (solution); 0–54.4 ppm (cad vapor)	470/580	16s	0.18 μM (solution); 3.22 ppm (cad vapor)
6 (MUS)	Biogenic amines	Colorimetric and fluorescent	Blue → green	0–125 μM (DMA)	385/478	≤11 s	3.58 μM
7 (HTBR)	Biogenic amines (e.g., cadaverine, putrescine, volatile amines)	Colorimetric and fluorescent	Rose red → yellow (daylight) non‐fluorescence → green (UV)	0–200 μM (DEA)	520/610	2 s	0.66 μM
8 (HYM)	Biogenic amines	Colorimetric and fluorescent	Yellow → pink (daylight) orange → non‐fluorescence (UV)	5–300 μM (spermine)	435/645	3 s	4.23 μM (spermine)
9 (CDT)	Biogenic amines	Colorimetric and fluorescence (OFF‐ON)	Weak fluorescence → red fluorescence colorless → yellow (daylight)	0–200 μM (TMA)	410/607	5S	5.47 μM (TMA)
10 (MTCA)	Cadaverine (biogenic amine)	Colorimetric fluorescence (AIE)	Orange‐red → green	0–30 μM	400/510→580	N.A.	0.34 μM
11 (HCPM)	Biogenic amines (especially cadaverine vapor)	Colorimetric fluorescence (AIE)	Orange red → green	0–30 μM (cad in solution); 0–30 ppm (cad vapor in solid state)	400/520→580	N.A.	0.45 nM
12 (2a)	Aliphatic amines (NH_3_, isopropylamine)	Colorimetric and ratiometric fluorescence	Orange red → green	0–100 μL	320/580→480	<1 s	22 nM (isopropylamine); 59 nM (NH_3_)
13 (CHO‐OH‐NO_2_)	H_2_S	Turn‐on fluorescence	Purple → blue	0–20 μM	685/718	30 min	0.75 μM
14 (NPS)	H_2_S	Turn‐on fluorescence	Colorless → yellowish green	0–20 μM	350/520	20 min	79 nM
15 (DPF‐NP)	H_2_S	Turn‐on fluorescence	Non‐fluorescence → orange	0–100 μM	380/590	3 min	96 nM
16 (DEM‐H_2_S)	H_2_S	Turn‐on fluorescence	Yellow → red	0–200 μM	505/710	30 min	80 nM
17 (XDS)	H_2_S	Turn‐off fluorescence (ICT)	Red → quenched (pink → pale yellow color change)	0–44 μM	535/611	12 min	0.98 μM
18 (WX‐HS)	H_2_S	Turn‐on fluorescence	Non‐fluorescence → orange‐red	0–15 μM	440/660	90 s	0.14 μM
19 (TPAT)	H_2_S	Turn‐on fluorescence	Non‐fluorescent → bright red	0–50 μM	500/670	2 min	0.738 μM
20 (TPAW)	H_2_S	Turn‐on fluorescence	Non‐fluorescence → red	0–100 μM	540/690	<10 s	7.45 μM
21 (HNMT)	H_2_S	Colorimetric and ratiometric fluorescence	Orange‐red → green	0–50 μM	400/505→565	N.A.	0.35 μM
22 (SL)	HSO_3_^−^	Ratiometric fluorescence	Bluish violet → blue	0–15 μM	520/810	40 s	38 nM
23 (Hy‐S)	SO_3_^−^	Turn‐off fluorescence	Yellow → colorless	0–35 μM (pH 4.0); 0–10.0 μM (pH 9.0)	462/515 (pH 4.0) 502/559 (pH 9.0)	60 s (pH 4.0), 30 s (pH 9.0)	1.19 μM (pH 4.0); 0.64 μM (pH 9.0)
24 (ZR‐I)	SO_3_^2−^/HSO_3_^−^	Turn‐on fluorescence	Weak → strong blue	0–90 μM	338/444	9 min	0.10 μM
25 (Sycy)	HSO_3_^−^ and volatile amines (DEA)	Colorimetric and fluorescent (turn‐off)	Red/Orange → colorless (HSO_3_^−^) orange → blue (amines)	0–50 μM (HSO_3_^−^); 0–300 μM (DEA)	625/650 (HSO_3_^−^); 480/611 (DEA)	150 s (HSO_3_^−^); 5 s (DEA)	3.50 μM (HSO_3_^−^); 7.11 μM (DEA)
26 (Dpyt)	HSO_3_^−^ and organic amines	Colorimetric and fluorescent(turn‐off for HSO_3_^−^, turn‐on for amines)	Blue → colorless (HSO_3_^−^); purple → blue (amines)	0–80 μM (HSO_3_^−^); 0–260 μM (propylamine)	596/658 (HSO_3_^−^) 590/660 (amines)	5 s (HSO_3_^−^); 4 s (amines)	7.34 μM (HSO_3_^−^); 2.23 μM (propylamine)
27 (HDXM)	HSO_3_^−^/SO_3_^2−​^ and BAs​	Colorimetric and fluorescent (turn‐off for HSO_3_^−^, turn‐on for amines)	Purple → colorless (HSO_3_^−^) red fluorescence quenched; purple → blue (amines) weak red → pink fluorescence	0–170 μM (HSO_3_^−^); 10–130 μM (Cad)	560/630 (HSO_3_^−^), 560/635 (amines)	3 s	1.02 μM (HSO_3_^−^/SO_3_^2−^)6.89 μM (CAD)
28 (Hx)	pH	fluorescent (ring‐opened/closed) Colorimetric and fluorescent (ring‐opened/closed) colorimetric and fluorescent (ring‐opened/closed) (ring‐opened/closed)	Green (521 nm) at alkaline, NIR (727 nm) at acidic	pH 2.56–8.78	460/521 & 727	N.A.	0.1 pH
29 (DIC)	pH	Turn‐on fluorescence	Colorless → cyan blue	pH 6.50–9.98	400/476	N.A.	N.A.
30	pH	Turn‐on fluorescence	Red (610 nm) strong at acidic, weak at neutral/alkaline	pH 5.0–7.0	530/610	N.A.	N.A.
31 (Mi‐Ly‐1)	pH	Colorimetric and dual‐emission fluorescent (pH‐dependent structural switch)	Green (560 nm) at acidic (pH 4.0–5.5); red (605 nm) at weakly alkaline (pH 7.5–8.5)	pH 4.0–8.5	420/560 (green) 550/605 (red)	N.A.	N.A.
32	N_2_H_4_	Turn‐on fluorescence	Dark orange → bright blue	0–30 μM	375/486	30 min	0.025 μM
33 (RP)	ATP	Turn‐on fluorescence	Colorless → purple	0–50 μmol/L	550/580	2 min	10.97 nM
34 (Resazurin)	Myoglobin Fe^2+^ (meat freshness)	Colorimetric	Blue → pink	0–0.7 mg/mL	601/570	30 min	0.051 mg/g

Abbreviation: N.A., not avaialable.

Despite significant advancements in small molecular fluorescent probes for food freshness detection, many challenges still exist. Accordingly, the rational design of fluorescent probes for food freshness monitoring must address several key challenges: (1) Insufficient coverage of target analytes: Current research is largely concentrated on high‐protein foods, with far fewer investigations into foods possessing complex matrices, thus preventing full and comprehensive detection coverage. Furthermore, only a very limited number of fluorescent probes are available for detecting flavor compounds in food. (2) Narrow target scope: Most fluorescent probes respond to only one spoilage marker, such as BAs, H_2_S, or SO_2_ derivatives. Multi‐target probes are still in early development, and the simultaneous specific detection of multiple BAs remains a major challenge. (3) Lack of predictive detection modes: Current probes function passively, assessing freshness only after spoilage has occurred, without enabling early prediction of spoilage or providing guidance on safe edible thresholds. As such, they cannot satisfy the demand for early prevention. (4) Insufficient practical performance: Fluorescent probes are often limited by factors such as operational complexity, response speed, portability, sensitivity, and cost, and thus cannot fully satisfy the practical requirements for diverse real‐world applications.

Combined with the current research status and existing problems, we believe that the future research on fluorescent probes for food freshness should focus on practicality, prospectiveness, and diversification, and focus on breaking through in the following directions to promote the industrialization and upgrading of the technology:

(1) Detection targets can be further diversified. More research should focus on complex‐matrix foods such as fruits, vegetables, cereals and processed foods. Attention should also be paid to the detection of flavor compounds in these foods. Based on the unique properties of different food matrices, we develop fluorescent probes with strong anti‐interference capacity and high adaptability to overcome the current bias toward high‐protein foods, thereby enabling comprehensive and accurate freshness evaluation across diverse food types.
(2) Efforts should be devoted to advance the development of multi‐target detection probes. Attention should be paid to the design of multichannel fluorescent probes for ^[74]^ simultaneous recognition of various spoilage markers, including BAs, hydrogen sulfide, and sulfur dioxide derivatives. Efforts should also target the challenge of specifically detecting multiple BAs simultaneously. Further, designing a single probe to enable multidimensional assessment of food spoilage will greatly improve detection efficiency and comprehensiveness.
(3) A prospective prediction system should be established. Researchers should integrate fluorescent probe detection data with food storage parameters (temperature, humidity, packaging, etc.) combined with emerging technologies such as big data and artificial intelligence. This enables the construction of food spoilage prediction models for active forecasting of food freshness and precise recommendations regarding safe consumption thresholds. Such a system allows early prevention and control of food waste and safety risks, driving the shift of detection strategies from passive detection to active early warning.
(4) The practical performance of such probes remains to be improved. Further optimize the design of fluorescent probes by simplifying detection procedures, shortening response times, enhancing detection sensitivity, and lowering synthesis costs. We should also promote deeper integration of probes with portable platforms such as smartphone‐based sensors and low‐cost test strips, and develop miniaturized, visual, and affordable detection systems suitable for diverse real‐world scenarios including on‐site rapid testing, household monitoring, and cold‐chain tracking. These efforts will facilitate the widespread application of fluorescent probe‐based freshness detection technologies.

## CONFLICT OF INTEREST STATEMENT

The authors declare no conflicts of interest.
